# Rapid Response of a Marine Mammal Species to Holocene Climate and Habitat Change

**DOI:** 10.1371/journal.pgen.1000554

**Published:** 2009-07-10

**Authors:** Mark de Bruyn, Brenda L. Hall, Lucas F. Chauke, Carlo Baroni, Paul L. Koch, A. Rus Hoelzel

**Affiliations:** 1 School of Biological Sciences, University of Durham, Durham, United Kingdom; 2 Climate Change Institute and Department of Earth Sciences, University of Maine, Orono, Maine, United States of America; 3 Mammal Research Institute, Department of Zoology and Entomology, University of Pretoria, Pretoria, South Africa; 4 Dipartimento di Scienze della Terra, Università di Pisa, Pisa, Italy; 5 Department of Earth and Planetary Sciences, University of California, Santa Cruz, California, United States of America; University of Chicago, United States of America

## Abstract

Environmental change drives demographic and evolutionary processes that determine diversity within and among species. Tracking these processes during periods of change reveals mechanisms for the establishment of populations and provides predictive data on response to potential future impacts, including those caused by anthropogenic climate change. Here we show how a highly mobile marine species responded to the gain and loss of new breeding habitat. Southern elephant seal, *Mirounga leonina*, remains were found along the Victoria Land Coast (VLC) in the Ross Sea, Antarctica, 2,500 km from the nearest extant breeding site on Macquarie Island (MQ). This habitat was released after retreat of the grounded ice sheet in the Ross Sea Embayment 7,500–8,000 cal YBP, and is within the range of modern foraging excursions from the MQ colony. Using ancient mtDNA and coalescent models, we tracked the population dynamics of the now extinct VLC colony and the connectivity between this and extant breeding sites. We found a clear expansion signal in the VLC population ∼8,000 YBP, followed by directional migration away from VLC and the loss of diversity at ∼1,000 YBP, when sea ice is thought to have expanded. Our data suggest that VLC seals came initially from MQ and that some returned there once the VLC habitat was lost, ∼7,000 years later. We track the founder-extinction dynamics of a population from inception to extinction in the context of Holocene climate change and present evidence that an unexpectedly diverse, differentiated breeding population was founded from a distant source population soon after habitat became available.

## Author Summary

In order to understand how biodiversity is generated and maintained over time, we need to understand the process by which populations form and diverge. Natural variation within species is typically partitioned among populations, which sometimes forms the basis for speciation events. One mechanism for the establishment of novel variation at the population level is through a response to emerging habitat. Here we use data from ancient DNA to show how elephant seal populations responded when new breeding habitat was gained and then lost over the course of approximately 7,000 years. We show that the seals quickly took advantage of newly available breeding habitat, far from the nearest extant breeding site, and that a highly diverse and genetically differentiated population was established over a matter of generations. The key factors were likely the abundant local food resource and extensive physical habitat that allowed rapid expansion after the initial founder event and a tendency for females to return to annual breeding sites in this species. Tracking the founder-extinction dynamics of historical populations provides insight into the likely implications of future environmental change. This is an important tool in our efforts to mitigate the impact of human-induced change.

## Introduction

Populations can respond to changing habitats by adapting (through natural selection or phenotypic plasticity), moving (to avoid habitat of reduced suitability, or take advantage of emerging habitat), by adjusting population size, or some combination of the above. Both natural selection and genetic drift can shape populations as they evolve in this context. It is unusual for there to be an opportunity to track population dynamics and genetics during extended periods of environmental change, but a now extinct population of elephant seals from the Victoria Land Coast (VLC) in the Ross Sea, Antarctica provides such an opportunity. Here we investigate the demographic and population genetic consequences of emerging breeding habitat suitable to this species in an environment rich in resources, and the impact of the eventual loss of that habitat.

Open beach along the VLC was probably released around 8,000–7,500 YBP, based on the grounding-line retreat of the West Antarctic ice sheet [1]. More recently, there is evidence that ice encroachment has taken place over the last 1,000 years at VLC, based on indications that Holocene raised beaches in several locations have been overrun by glacial expansion [2]–[4], and that these same beaches show evidence of being formed under wave-dominated conditions characterized by reduced sea ice [5]. Today the region is mostly enclosed by year round land-fast sea ice, and is therefore unsuitable for southern elephant seal breeding. Elephant seals are not found along the VLC today.

**Figure 1:**
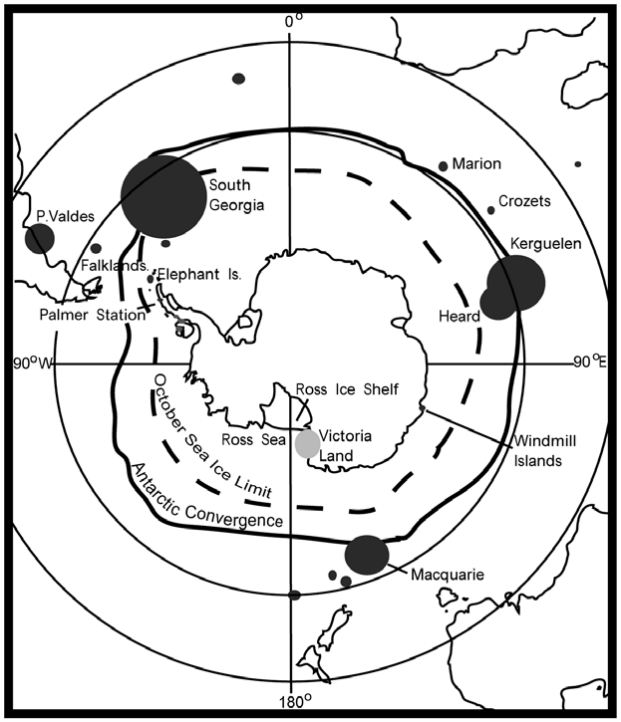
Map of study region showing major and minor southern elephant seal (*Mirounga leonina*) breeding colonies. With the exception of VLC (shown in gray), size of the circles indicates relative colony size.

Southern elephant seals have a circumpolar distribution, breeding on sub-Antarctic (north of the Antarctic convergence) and Antarctic (south of the Antarctic convergence) islands (Figure 1). They are annual breeders, showing fidelity to a set of established breeding colonies, and spend the non-breeding seasons foraging over large distances at sea, and hauled out on beaches for moulting [6]. The same beaches used for breeding are often used for moulting, but other locations may also be used, especially by adult males, sometimes including the Antarctic mainland [6]. Temperate continental breeding habitat historically included South Africa and Tasmania, but is now restricted to Peninsula Valdés, Argentina [6]. Small colonies are found on open beaches at Palmer Station and in the Windmill Islands (probably originating from the Macquarie and Heard Island colonies [7]) close to the Antarctic continent, but the presence of southern elephant seals along the Antarctic coastline in modern times is primarily on foraging excursions that originate from sub-and/or Antarctic breeding colonies or haul-out sites [8]. There are no examples of elephant seal breeding colonies where it is necessary to cross ice to reach the site from open water [6], possibly because the energetic expense would be too high for competing, polygynous seals that fast during the breeding period.

A large number of exceptionally well-preserved skin samples and mummified remains, including pups, were collected from raised Holocene beaches along the VLC, adjacent to the Ross Sea (Figure 1), from a period when the beaches were exposed to open water [1],[9]. The vast quantity of seal remains and their extensive geographical coverage (Table S1) suggests that the VLC was not merely a moulting site, though breeding could have been a minor or temporary aspect of site usage. To investigate this further, and to understand the evolutionary history of this putative population, we used strict ancient DNA (aDNA) protocols [10] to amplify and sequence 325 bp of the most variable part of the mitochondrial DNA (mtDNA) control region. Direct radiocarbon dating was used to date 223 of these samples (Table S1).

We utilise these data to track the founder-extinction dynamics of an ephemeral population from inception to extinction in the context of Holocene climate change. We test the hypothesis that the release of new habitat in a highly productive environment can lead to the rapid founding and expansion of a new population, and investigate the consequences with respect to genetic diversity and gene flow among populations. We further investigate the consequences of the loss of this habitat on genetic diversity and connectivity.

## Results

**Figure 2:**
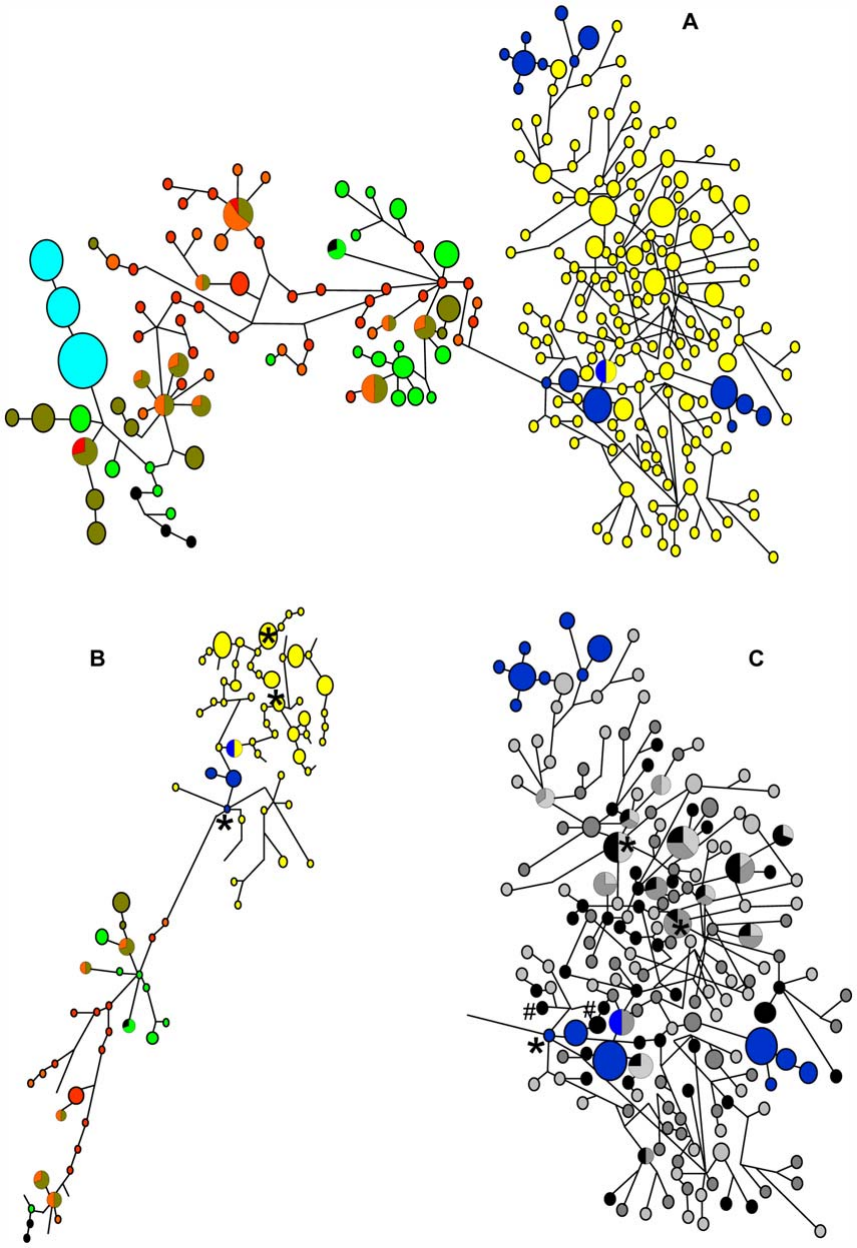
Networks of phylogenetic relationships among ancient and extant mitochondrial DNA haplotypes. (A) Median-joining network where the size of the circle indicates relative frequency of the haplotype. Yellow – Victoria Land Coast (VLC); dark blue – Macquarie Island (MQ); brown – Falklands; orange – Elephant Island; green – Marion Island; light blue – Peninsula Valdés; red – South Georgia; black – Heard Island. South Georgia and Heard Island relationships are based on 240 bp of mtDNA (see Materials and Methods). Networks were post-processed to reduce complexity using the Maximum Parsimony option in Network 4.5 [13]. (B) Network showing torso only, illustrating major connections among basal haplotypes. Image has been rotated to fit the page. Samples coloured as in part ‘A’. Likely VLC founder haplotypes, based on rho (*ρ*) estimates, indicated by ‘*’. (C) MQ and VLC clade only, illustrating relationship between MQ haplotypes and the age of the VLC samples. Blue: MQ; light gray: VLC<1500 YBP; dark gray: VLC 1501–3000; black: VLC 3000+. Likely VLC founder haplotypes, based on rho (*ρ*) estimates, indicated by ‘*’. Nearest VLC neighbours of the proposed VLC-founder haplotype (sampled from MQ) indicated by ‘#’.

Ancient DNA extractions were successful for approximately 90% of samples trialled. From 223 successfully sequenced ancient VLC sample extractions, 177 haplotypes were identified, whereas from 49 MQ samples, 16 haplotypes were identified (Table 1; GenBank accession numbers: FJ168073–FJ168343). A single haplotype was shared between VLC and MQ. Summary details for all other extant colonies are presented elsewhere [11],[12]. To determine relationships between the VLC seals and extant seal colonies, we used various phylogeny reconstruction methods [13],[14]. While a large degree of reticulation was evident for the VLC samples (Figure 2), all methods unambiguously defined two major lineages; the ancient VLC samples together with extant samples from MQ, and a lineage including all other extant colonies (Figure 2; only median-joining network reconstructions are shown). Bayesian analyses of the ancient VLC data, incorporating an explicit post-mortem damage model [15], calibrated against the estimated calendar age of the ancient samples (Table S1) produced a mtDNA hypervariable region (HVR) rate estimate of 9.80×10^−7^ (Figure S1; 95% highest posterior density interval (HPDI) 1.67×10^−9^–2.06×10^−6^) substitutions per site per year (s.s.yr^−1^). This rate is in agreement with estimates obtained from various other aDNA datasets [16] including a close match with an HVR rate of 9.6×10^−7^ s.s.yr^−1^ calculated over a similar timeframe (6,424 YBP) from Adelie penguin aDNA [17], and with the mean human HVR pedigree rate estimate derived from a meta-analysis (9.5×10^−7^ s.s.yr^−1^) [18]. Although HPDIs were broad, the sampling distribution returned a well-resolved peak with strong bounds (Figure S1). After [19]–[21], uncertainty in the actual substitution rate estimate was not incorporated into further analyses, in order to isolate uncertainty in the coalescent process. In the context of the well-documented geochronology of the VLC [1]–[5],[9],[22], our results applied in further analyses (e.g. IM; mismatch distributions – see below) provide strong support for the HPD rate estimate, and for the similar molecular rates calculated from other species over Holocene timeframes and from pedigree data [16]–[18],[23],[24]. Although controversial [25],[26], as they are an order of magnitude or more faster than ‘traditional’ substitution rate estimates derived from interspecific phylogenetic datasets or the fossil record [24], these high intraspecific estimates have been quite consistent and are now well supported [16]–[18],[23],[24],[26].

**Table 1:** Population genetic summary and demographic statistics for Victoria Land Coast (VLC) and Macquarie (MQ) populations.

	*N*	*S*	*h*	*H_d_*	*k*	*S/k*	*Fs*	*D*	BF
**VLC**	223	91	177	0.996 (0.001)	7.73	11.77	−323.00	−1.66	exp*
							*P*<0.01	0.10>*P*>0.05	
**MQ**	49	23	16	0.915 (0.019)	6.58	3.50	−0.11	0.88	con*
							*P* = 0.13	*P*>0.10	

*P* values were obtained with 50,000 coalescent simulations. *N*: number of individuals; *S*: segregating sites, *h*: number of haplotypes; *Hd*: haplotype diversity (±s.d.); *k*: average number of nucleotide differences; *S/k*: ratio of *S* over *k*; *Fs*: Fu's *Fs*; *D*: Tajima's *D*; BF, approximate Bayes Factor selection of alternative demographic models (constant vs. exponential), using the difference in harmonic means of sampled marginal likelihoods implemented in BEAST [58]. * indicates 3<BF(±s.e.)<10.

Founder analysis identified three closely related haplotypes within the MQ/VLC clade as potential founders (Figure 2B), based on minimum *ρ* value estimates for that clade. Two of these were sampled from the VLC site, while one was sampled from MQ. Coalescent theory predicts that ancestral haplotypes will be basal and/or central within the haplogroup [27],[28], as is the case for these haplotypes. Mapping the estimated calendar ages of the VLC haplotypes onto the MQ/VLC network supported these results, as the potential MQ founder haplotype's nearest-neighbours date to early in the VLC colonies' history, as do the potential founder haplotypes sampled from the VLC (Figure 2C). In addition, minimum inter-population *ρ* value estimates identify MQ as the most likely source of the VLC samples (Table 2).

**Table 2:** *ρ* distances (±s.d.) between the mtDNA pools of Victoria Land Coast (VLC) samples and those of potential source populations, representing all major extant southern elephant seal breeding colonies.

	MQ	SG	HD	MA	EI	FL	PV
**VLC**	0.2 (0.2)	1.6 (0.14)	3.0 (0.25)	3.5 (0.5)	3.5 (0.5)	6.86 (0.86)	20.16 (1.67)

VLC: Victoria Land Coast; MQ: Macquarie Island; SG: South Georgia Island; HD: Heard Island; MA: Marion Island; EI: Elephant Island; FL: Falklands; PV: Peninsula Valdés. Calculations for SG and HD were based on 240 bp of sequence data (see Materials and Methods), and are likely underestimates.

Coalescent estimates [29] of the time of splitting for MQ and VLC (t = 6,167 YBP; 90% HPDI = 5,116–7,674 YBP; Figure S2; Table S2) corresponds closely with the mid-Holocene retreat of the Ross Sea ice sheet from the VLC (7,500–8,000 YBP) [1], and thus the consequent opening of newly available seal habitat, and coincides with the age of the oldest ancient sample (7,087 YBP; Table S1). On this basis, colonisation of the VLC likely began prior to 7,000 calendar YBP.

**Figure 3:**
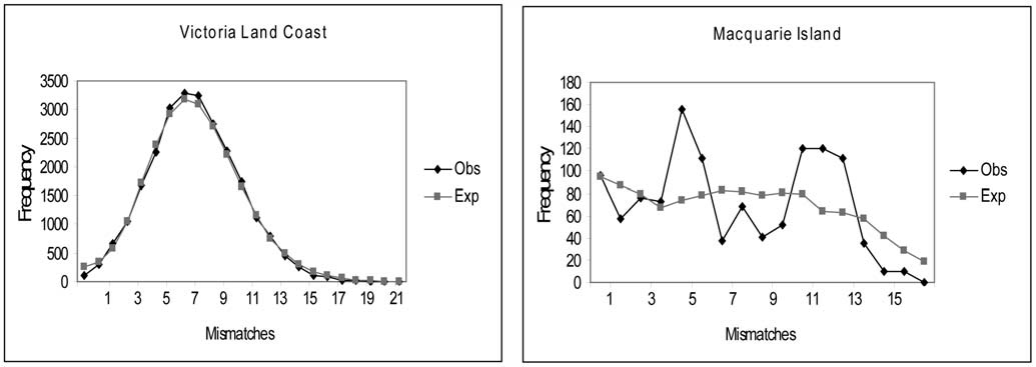
Mismatch distributions for mitochondrial DNA haplotypes sampled from the VLC and MQ populations.

Bayesian demographic model selection and population genetic and coalescent-based statistics provide a signature of subsequent expansion in the VLC population, while MQ shows a signature of a long-term stable population (Table 1). Similarly, mismatch distributions [30] show a strong signal for expansion in the VLC population (with tau = 5.48; mean expansion time estimate = 8,600 YBP (95% CI = 7,185–10,298 YBP)), while the mismatch distribution for MQ is multi-modal and therefore consistent with long-term stability (Figure 3). FLUCTUATE [31] analyses indicated strong growth in VLC samples from ≥3,001 YBP (g = 648±44 sd), while 1,500–3,000 year old samples (g = 254±33) and the most recent samples (<1,500 years old; g = 119±14) showed lower growth rates.

Recent back migration to MQ can be estimated by examining for the presence of MQ lineages that are most likely to have evolved at VLC (Figure 2), that is, MQ haplotypes that are derived from VLC haplotypes [27]. It is clear from the network that this is the case for a number of MQ haplotypes, and again mapping the ages of their nearest VLC neighbours suggests increasing recent (range of nearest-neighbour dates: 506–1,565 YBP) contact from VLC to MQ (Figure 2C). Consistent with this notion of some MQ haplotypes being derived from VLC haplotypes, φ_ST_ values appear to decrease over time between VLC and MQ (>3,000 YBP: φ_ST_ = 0.208; 1,500–3,000 YBP: φ_ST_ = 0.161; <1,500 YBP: φ_ST_ = 0.149; *c.f.* Table S3). Comparing VLC to other modern populations, φ_ST_ showed the opposite trend (>3,000 YBP: φ_ST_ = 0.458; 1,500–3,000 YBP: φ_ST_ = 0.539; <1,500 YBP: φ_ST_ = 0.560). One possible interpretation would be increasing contact between VLC and MQ. These significant φ_ST_ values also show that VLC must have become an independent breeding population, and was not merely or predominantly a geographically distant moulting haul-out site. Results using an isolation with migration model [29] provide additional support for post-founder unidirectional migration from VLC to MQ only (m1 (into Macquarie) = 0.45, 90% HPDI = 0.07–1.79; m2 (into VLC) = 0.005, 90% HPDI = 0.005–0.115), and an estimated time of migration of 1,365 YBP (90% HPDI = 565–4,325 YBP) (Figure S2, Table S2). Based on the age of the youngest seal remains recovered, final abandonment of the VLC site likely took place around 400 YBP (Table S1).

**Figure 4:**
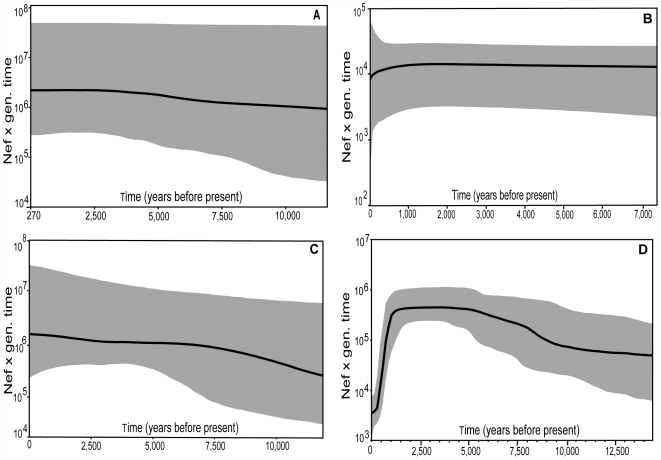
Bayesian skyline plots of effective population size change through time. The black line indicates the median posterior effective population size through time. The gray lines illustrate the 95% HPDI, taking into account coalescent model and phylogenetic uncertainty, for: (A) VLC alone; (B) MQ alone; (C) VLC and Marion Island combined; (D) VLC and MQ combined.

Bayesian skyline plots [32], which simultaneously estimate both the timing and magnitude of effective (female) population size change (*N*e), provided more equivocal results. There is only a slight indication of expansion when VLC is considered on its own (Figure 4A), and the 95% HPDIs are broad and overlapping. Considering MQ on its own suggests a stable population at an estimated *N*e that is substantially lower than for VLC (Figure 4B). We then combined VLC with extant populations, first with Marion Island where the census population size is known to be similar to that on MQ [6],[33],[34], and for which there is little evidence of connectivity with either MQ or VLC ([11]; see above). In this case there is a stronger indication of expansion, but no indication of a population decline (Figure 4C). A very similar result was found when we combined VLC and Elephant Island (BSP not presented), which also has a similar or smaller census size to MQ [6],[35]. Finally, we combine VLC with MQ (Figure 4D). Here we see a trace that indicates both population expansion and decline, at dates roughly consistent with our previous estimates for these events.

We then assessed levels of diversity from two time periods for the VLC, and for the modern MQ, though our VLC samples from the relatively recent past come mostly from the beginning of the apparent decline period (VLC samples>1,000 YBP: π = 0.0243±0.0001, *r* = 44.7, *n* = 163; VLC<1,000 YBP: π = 0.0224±0.0012, *r* = 41.7, *n* = 60; MQ: π = 0.0202±0.0013, *r* = 15.0, *n* = 49; *r* = haplotype richness, corrected for sample size). Only the VLC>1,000 YBP and MQ samples had non-overlapping distributions for π according to the analysis in DNASP (see Materials and Methods). The haplotype network (Figure 2) and relative haplotype richness values for VLC and MQ illustrate the large difference in diversity, which may in part be due to diversity being lost during 19^th^ century sealing at MQ. However, there is no signal for a population bottleneck and subsequent expansion at MQ (Figure 3), and the level of diversity there is comparable to that seen at other islands [11].

Estimates of *N*e derived using IM indicate a large effective population size at VLC (219,255; 90% HPDI = 187,888–267,582), and much lower estimates for MQ and the putative ancestor population (MQ: 6,727 (2,566–11,969); Ancestral: 13,263 (9,320–22,827)) (Figure S2, Table S2). This may suggest continuity between the ancestor and MQ populations. Note that un-sampled (‘ghost’) populations may inflate the apparent size of the ancestor population in this two population model [29]. The larger size estimate for VLC is consistent with the skyline plot results (Figure 4). Calculating the time to the most recent common ancestor (TMRCA) using Bayesian phylogenetic methods [14] showed similar overlapping age estimates for the VLC and MQ samples (VLC: 11,740 YBP; 95% HPDI = 9,097–14,710; MQ: 11,090; 95% HPDI = 8,461–14,030), pre-dating the founding of VLC. All other modern populations considered both separately and pooled gave a younger TMRCA estimate, which approximated the time when the VLC site was founded (‘Modern’ pooled: 5,570 YBP (3,833–7,189); Figure S3).

## Discussion

Effective management and mitigation of the impact of climate change on biodiversity requires an understanding of how populations are likely to respond to the consequent gain and loss of critical habitat. Predictive models [e.g. 36],[37] typically base expectations on extant population distributions. However, investigating the dynamics of populations during past climate change provides a resource on which future inference can be more accurately based [38]–[40]. In this study the impact on habitat was driven by climate, as it involved the retreat and subsequent advance of both glacial and sea ice. The elephant seal, like many other large, highly mobile marine species, travels great distances during foraging excursions, whereas population structure is seen on a smaller geographic scale [11].

One of our key hypotheses was that a highly mobile species can respond to distant habitat gain and loss through migration. All potential breeding habitat on the Antarctic mainland for this species was likely under ice during the last glacial period, so the VLC population must have been established sometime after the last glacial maximum. Geological evidence suggests that the timing of this would have been possible around 8,000 YBP [1]–[5],[22]. Our results indicate that the VLC population is most closely related to MQ among the extant populations. This was supported by the phylogenetic reconstructions and inbreeding coefficients. Rho statistics also show MQ to be the most likely source for the founding population at VLC under this scenario. However, while we assume that VLC must have been founded from an extant sub-Antarctic population, there are aspects of the phylogenetic reconstructions that may suggest alternative interpretations. First, the level of diversity at VLC is higher than that of the putative source population, second, a number of the MQ haplotypes sampled appear to be derived from VLC haplotypes, and third, the TMRCA for MQ and VLC is older than the splitting time.

We therefore investigated this aspect of the relationship between VLC and MQ in considerable detail. One possible interpretation is that both VLC and MQ were derived historically from a third population. However, all major extant populations were sampled for this study, and none are possible candidates due to their genetic distance from the VLC. Therefore, this hypothetical population would have to be extinct today. The only known possible candidate would be the population from the north-western coast of Tasmania, extinct since prehistoric times [41]. Our only extant example of a mainland population is depauperate of mtDNA variation (on Punta Norte, Peninsula Valdés, Argentina, see Figure 2), and Tasmania is 1,500 km further from VLC than MQ (raising questions as to why the VLC population would be founded by the Tasmanian rather than by the geographically closer MQ population). Neither of these considerations rule out this possibility, but other factors may.

If both VLC and MQ had been derived as founders from an unsampled historical population, both should show evidence for expansion, though only VLC does (based on Bayesian demographic model selection, coalescent assessments from Fluctuate analyses, neutrality statistics, and the mismatch distributions; see Table 1). Also, if they split separately from a common ancestor, why should an estimated splitting time between VLC and MQ coincide with the likely date that habitat was released along the VLC, when the ice sheet was retreating? These observations also count against the notion that MQ was founded from VLC, which is in any case made highly unlikely due to the lack of available habitat at VLC more than 7,000–8,000 years ago.

We propose instead that VLC was founded by MQ, and that some seals from a declining VLC population returned to MQ. These interpretations are supported by the following. If a population is founded from another, there should be haplotypes in the source population that are basal within a lineage that includes both populations. This is true for the VLC and MQ populations, though the most convincing case is for just one, infrequently sampled haplotype in MQ found at the division point between the VLC/MQ lineage and the rest of the populations (see Figure 2B). This haplotype also has a close relationship to the two VLC-sampled potential founder haplotypes. There is also a basal haplotype within the network that is shared between MQ and VLC (Figure 2B), suggesting a common root for the two populations. In further support of this is the illustration that relatively old VLC haplotypes within the network are closely allied with MQ basal haplotypes (see Figure 2C). Even so, it is clear that basal haplotypes are not very well sampled from the MQ population. One possible explanation could be the loss of lineages at MQ in the relatively recent past (perhaps as a consequence of sealing pressure) and other sampling effects.

There is a signal from the IM analyses indicating unidirectional migration from VLC to MQ at about 1,300 YBP. This is consistent with the idea that seals from VLC returned to MQ as the VLC population was starting to decline. A trend for decreasing genetic distance between these populations over time may also support this (though unlike the various non-equilibrium analyses used, the φ_ST_ data assume equilibrium conditions that probably don't apply). From the phylogenies, you would expect to see some MQ haplotypes as derived tips within the network, and this pattern is clearly evident. Furthermore, these are primarily derived from the more recent VLC samples (dated to 506–1,565 YBP; see Figure 2C). The IM estimates depend on an accurate substitution rate estimate, which from our calculations have a large error associated with them. However, the HPD rate estimate we derived is very similar to estimates derived from intraspecific analyses for a variety of taxa [16]–[18],[23],[24],[26],[38], and in all cases where analysed (as in this study), these rates provide good correspondence to the timing of known historical and geologic events (e.g. [38]–[40]). It is in that context that we apply this substitution rate.

A further consideration with IM is the assumption that the two populations being compared were sampled at the same point in time. While this is not the case for comparisons between VLC and MQ, the chronological results from this analysis are nevertheless consistent with those obtained using other methods and with the geologic record. This also applies to other analyses that involve the incorporation of different chronologies into estimates of population differentiation (such as φ_ST_) or population expansion (such as Tajima's D) [42]. We tested the robustness of these comparisons for VLC sample subsets involving timeframes that represent a small proportion of the TMRCA (<10%), and show that our conclusions remained consistent (Table S3).

Further support for the proposed pattern of demographic expansion and contractions at VLC comes from the BSP profile shown in Figure 4D. This BSP plot shows population expansion and contraction signals consistent with all other data from the genetic analyses, and with the expected timing based on geologic inference [1]–[5],[22]. The problem is that the construction of this plot violates some key assumptions. First, there is an assumption of panmixia, and the Φ_ST_ data (for example) show that MQ and VLC are differentiated. However, empirical data and simulation studies suggest skyline plots are robust to violations of assumptions of panmixia [19],[20],[32],[38]. There is also the potential problem associated with combining populations that have very different effective sizes. However, we test this by combining VLC with other extant breeding populations (Marion Island; Elephant Island) with very similar or smaller census sizes as that for MQ ([6], [33]–[35]; see Figure 1 and Figure 4C). In this case there is no decline seen at 1,000 YBP, or at any other point. Further, it isn't clear why the combination of VLC and MQ should show a decline as old as 1,000 YBP, nor why the end point should be lower than the estimated effective population size on MQ (see Figure 4B and 4D). We suggest that this could be a result of VLC seals having returned to MQ, and taken with them lineages that retain the signal of the VLC population crash. The profile for VLC alone may not show this crash due to the paucity of very recent samples from this site (see Table S1). However, the various problems discussed mean that this interpretation should be treated cautiously, and only as consistent with the stronger inference gained from the various other analyses.

As mentioned previously, the greater diversity of the VLC site was unexpected for a founder population, and much of it is unique to that population. Two key factors may be important. First, the founded population could have established and grown quickly, if as now the forage resource was high. Second, high numbers were supported over time (estimated in this study as at least an order of magnitude higher than a long-term stable population size estimate for MQ). Again, this likely reflects a large (hundreds of kilometres of newly available habitat [22]) local environment rich in resources for the VLC population. The greater diversity at VLC compared to MQ may also reflect more rapid lineage extinctions at MQ, where *N*e was lower (and perhaps impacted by sealing pressure on population size during the last century [43]). Given that the mtDNA genome includes numerous functional loci, selection needs to be a consideration when interpreting these data. However, in this case the high diversity and lack of evidence for increased frequency of specific haplotypes in the VLC mean that there is no clear evidence to support that interpretation.

Another factor is the relatively old TMRCA for MQ and VLC, however this is expected if they share common ancestry (as they would if one was derived from the other) and if some level of gene flow was maintained (as is suggested by the IM analysis). The more recent, shared TMRCA among the other populations is perhaps more unexpected (see Figure S3). One possible explanation is that the environmental changes that led to the founding of VLC also had a demographic impact on the other sub-Antarctic breeding sites, though this would need to be investigated further to be confirmed.

Taken together, our results indicate that new habitat was quickly exploited, and that the founder population became independent of the distant source population (given high and significant φ_ST_ values throughout the relevant time period, the distinct mismatch distribution profile, and IM results for time of splitting). The genetic data clearly distinguishes the VLC population from a site used exclusively for moulting, which could have represented the temporary assemblage of seals from one or more separate breeding populations. The implication is that discovery of emergent habitat was facilitated by long-distance foraging excursions, and that some level of continued dispersal between sites allowed the return of a proportion of the founder population to the source colony, when the new habitat was lost many generations later. This type of long distance networking may be possible for other species of conservation concern at times of rapid environmental change. In contrast, species with a more local distribution and range may be less able to cope. For example, Arctic foxes from midlatitude Europe went extinct during the Pleistocene when available habitat contracted [44]. Predictive modelling for the development of conservation strategies should therefore take these factors into account. In general, these data indicate the rapid establishment of a genetically differentiated population in emerging habitat, where diversity levels remained very high. Much of this novel diversity was later lost when the VLC breeding habitat was lost. This suggests that when conditions are optimal, the potential for future adaptive radiation over relatively short time frames is high, and that this potential can be lost again just as rapidly.

## Materials and Methods

### Sample collection, ancient DNA extraction, PCR, and sequencing

Southern elephant seal skin samples were collected from beaches along the Victoria Land Coast, Ross Sea, Antarctica (Table S1), and stringent sample collection procedures were followed to avoid anthropogenic- or cross-contamination of samples. Ancient DNA extractions and PCR setup were conducted in a dedicated aDNA laboratory at the University of Durham. Strict aDNA protocols were adhered to [10], and results carefully assessed in this context: 1) One-way movement of personnel/ equipment/ PCR set-up from aDNA lab to Molecular Ecology lab; 2) Regular bleach & UV treatment of all work surfaces; 3) Use of aerosol-resistant pipette filter-tips for aDNA work; 4) Regular autoclaving of all equipment, plasticware, and DNase & RNase-free deionized sterile water from a dedicated aDNA autoclave; 5) Use of multiple blank controls for both extraction and PCR procedures; 6) Replication, cloning and independent replication of a subset of aDNA samples [22]; 7) Confirmation that results make phylogeographical sense.

To check the accuracy of the extraction, PCR, and sequencing steps, approximately 30% of samples were replicated for all steps, while 10 samples were replicated 4 times. These steps exceed established replication protocols for the aDNA field [10],[45]. Nine samples extracted independently in a different lab five years prior to the current study by another researcher for an earlier study [22] were re-extracted and sequenced (i.e. independent replication), and all sequences matched. Six PCR products were cloned (six clones from each product were sequenced) to evaluate template damage and to check for nuclear pseudogenes and contamination.

Skin samples were digested in 3 mL of extraction buffer, containing 0.5% SDS, 0.45 M EDTA (pH 8), 10 mM Tris-HCL, and 50 µl Proteinase K, at 50°C overnight. Digests were centrifuged at 13,000 g for 1 min, the aqueous phase was removed, and added to 5 volumes of PB Buffer (QIAgen) and vortexed. This solution was passed in 700 µl quantities through QIAquick spin filter columns (QIAgen), centrifuged at 13,000 g for 1 min, and the flow-through discarded. DNA was then twice washed with 700 µl of Buffer PE (QIAgen), centrifuged as before, and the flow through discarded. The filter column was placed in a sterile 1.5 ml tube, and DNA was eluted by applying 35 µl of Buffer EB (QIAgen) to the column. This was then centrifuged at 13,000 g for 1 min. DNA samples were stored at −20°C until required. DNA extractions were performed in batches of 20 samples with 1–2 blanks (no tissue) per batch.

PCR reactions (25 µl volumes) contained: 2 µl extract, 1 U Platinum *Taq* Hi-Fidelity, 1×buffer, 1.5 mM MgCl_2_ (Invitrogen Ltd., UK), 0.2 µM each primer and 200 µM each dNTP. BSA was found to be inhibitory, possibly due the presence of hairs in the elephant seal skin samples [46]. Thermal cycles were as follows: 95°C for 2 min, 45 cycles of 94°C for 45 sec, 52–55°C for 45 sec, and 72°C for 45 sec, followed by a final extension at 68°C for 10 min. Negative controls were used for all PCR runs. Positive PCR amplicons of the correct size were purified using a QIAgen Purification Kit according to manufacturer's instructions. Overlapping fragments of DNA were amplified for a final target of 325 bp of the most variable portion of the mitochondrial control region (HVR1). Primers used: SESanc3f 5′-GCTGACATTCTACTTAAACT-3′ & SESanc3r 5′-ATGTACATGCTTATATGCAT-3′; SESmdbf 5′-AGCCCTATGTATATCCTGCATT-3′ & SESmdbr 5′-CAGTATAGAAACCCCCACATGA-3′. Purified PCR products were sequenced at Macrogen (S. Korea) and at the University of Durham. There was no variation among replicates for a given sample. During cloning and sequencing, no evidence of nuclear copies was detected, while one sample displayed evidence of damage and was removed from further analyses, consistent with the generally excellent preservation of these ‘freeze-dried’ Antarctic samples.

### ^14^C Radiocarbon dating of ancient tissue and calibration methods

Radiocarbon dating of 223 ancient VLC samples was by Accelerator Mass Spectrometry (AMS) at the NOSAMS laboratory at Woods Hole Oceanographic Institution and at the University of Arizona. All dates were calibrated using the CALIB [47] program and the Marine04 dataset, with a time-dependent Southern Ocean marine-reservoir effect (on average, delta-R = 791±121 yrs), derived from paired radiocarbon and uranium-thorium dates of Holocene corals collected along the VLC (BL Hall et al. unpublished data). Radiocarbon dates were converted to calendar dates (Table S1).

### Modern DNA extraction, PCR, and sequencing

Modern southern elephant seal samples were analysed after completion of all aDNA work (Macquarie Island *n* = 49; Marion Island *n* = 48) or data incorporated from earlier studies using Genbank sequences (Peninsula Valdez, *n* = 50; Falklands, *n* = 56; Elephant Island, *n* = 30; South Georgia, *n* = 30; Heard Island, *n* = 6) [11],[12]. All major extant southern elephant seal breeding colonies are represented in our analyses (Figure 1). Modern sequences were aligned to the aDNA sequences and trimmed to create a final combined HVR1 dataset of 269 modern and 223 aDNA sequences of 325 bp in length; except for the South Georgia and Heard samples, for which only 240 bp of homologous sequence data were available. Tissue digestions followed the protocol for aDNA samples, followed by standard phenol: chloroform extraction [48]. Blank controls were incorporated. PCR setup was the same as for aDNA, except that the number of thermal cycles was reduced to 35, and annealing temperature was set at 55°C. Primers (SESM1F: 5′-TGACATCCATCCCCCTTATT-3′ and SESMR: 5′-TGTGTG ATCATGGGCTGATT-3′) were designed to amplify a 440 bp fragment encompassing the entire aDNA target fragment.

### Phylogeographic and demographic analyses of ancient and modern sequences

Phylogenies [13],[14] were constructed from the full dataset to visualise relationships among modern and ancient haplotypes. Median-joining networks were implemented in Network 4.5 (http://www.fluxus-engineering.com) [13] based on 325 bp sequences for the full dataset, and a second network was generated focussing on the MQ/VLC clade. Additional analyses were also conducted with a reduced dataset of 240 bp incorporating South Georgia and Heard Islands samples. No pre-processing was required, but Maximum Parsimony (MP) post-processing was implemented, to remove links not used by the shortest trees in the network, thereby reducing network complexity. Networks were analysed according to founders' analysis principles [27]. As a consequence of the close genetic relationship identified between MQ and VLC and initial founders' analysis results, we also adopted criteria ƒ0–2 proposed by Richards et al. [27] to distinguish between candidate founders and back-migration. In brief (see [27] for details), potential founders are not permitted at the tips of the clade to minimise the possibility of recurrent mutations being designated founders, while back-migration can be identified as haplotypes derived from haplotypes intermediate to those of the founder. Ancestral haplotypes for the MQ/VLC clade were identified on the basis of minimum *ρ* value estimates [49] for that clade [24]. In addition, calendar ages (derived from radiometric ages) were mapped onto the MQ/VLC network to determine whether founder haplotypes were consistent with haplotype ages along their respective lineages. Minimum inter-population *ρ* value estimates were then calculated between ancestral VLC haplotypes and the seven modern population haplogroups, representing all major extant breeding colonies (potential source colonies). An implicit assumption in these analyses is that no alternate potential source population (and its' mitochondrial lineages) has gone to extinction, and thus remains unsampled in the current study.

Bayesian coalescent analysis using Markov Chain Monte Carlo (MCMC) was implemented in the program BEAST v. 1.4.8 and v. 1.5.b [14], using a substitution rate model (GTR+I+G) determined by MODELTEST [50]. Phylogenetic relationships among all samples were also investigated in BEAST through the construction of a Maximum Clade Credibility Tree. Version 1.5.b was provided by one of the BEAST program developers (Andrew Rambaut), and incorporates a post-mortem damage model [15], which takes into account the potential for sequence damage to influence the outcome of the aDNA analyses. Demographic inferences were essentially the same with or without the incorporation of the post-mortem damage model (details available from authors). BEAST analyses used MCMC to integrate over all credible genealogies, while simultaneously estimating both substitution rate (using corrected calendar ages as calibrations) and the demographic history of the sampled sequences. This was done under three demographic coalescent models: constant, exponential, and Bayesian Skyline Plot (BSP [32]). Serially sampled aDNA sequences provide the opportunity to calibrate substitution rate directly [17]. Rate estimates for further analyses were derived under the BSP model, from the VLC samples only (strict clock model), as genetic subdivision (i.e. population structure) within a dataset may bias rate estimates upwards [51]. A BSP coalescent model is preferable for rate estimation as it ‘averages out’ the demographic history of the sample, which can be considered a nuisance parameter in this instance [32]; notwithstanding, substitution rate estimates have been shown to be consistent across alternate underlying demographic coalescent models [16].

To ensure the substitution rate estimate was not merely an artefact of the dates provided, i.e. that the aDNA sequences used in this study were rate informative, these analyses were repeated three times by randomly re-assigning calendar ages to sequences. A recent re-analysis [51] of exceptionally fast rates reported for tuatara [52] utilised this approach to show that the tuatara aDNA dataset was not rate informative. For our study, rates estimated from the re-assigned ages were in all cases highly skewed to zero (Figure S4), while the rate estimate derived from the ‘true’ calendar ages returned a bell curve estimate not encompassing zero (Figure S1). This test indicates that the sequences were informative. Three alignments were run in BEAST: 1. Full dataset - all samples; 2. Ancient samples only (VLC); 3. VLC and MQ clade (BSP model only). As a control measure to determine whether the MQ/VLC BSP would be influenced primarily by the smaller effective population size of MQ relative to that of the VLC, we generated a further two BSPs by including VLC with two modern populations, Elephant Island and Marion Island, respectively, each with similar or smaller population sizes to MQ (Figure 1) [6], [33]–[35]. Although combining separate populations violates the assumption of panmixia, simulation and empirical studies have shown skyline plots to be robust to violations of this assumption [19],[20],[32],[38]. Three independent MCMC samples per alignment were run for 20,000,000 generations, sampled every 2,000 generations, after the initial 10% were discarded as burn-in. These three independent samples were combined using LogCombiner and analyzed in Tracer v1.4 (both programs distributed with BEAST), to generate credibility intervals that represent coalescent model and phylogenetic uncertainty, and to produce the final BEAST results. The effective sample size (ESS) for all parameter estimates was at least 200, while the autocorrelation times of MCMC plots indicated that runs converged on the equilibrium distribution.

Summary and population genetic statistics were calculated in DNASP v. 10.4.9 [53]. The number of segregating sites *S* over the average number of pairwise nucleotide differences *k* describes the ‘expansion coefficient’ [54], with high values indicating an increase in population size through time, while low values should be indicative of relatively long-term constant population size. DNASP was also used to compare genetic diversity estimates for nucleotide diversity. We used 1,000 coalescent simulations to derive 95% confidence intervals for these estimates, in order to assess whether the distributions overlapped (with non-overlapping distributions providing a conservative estimate of significant differentiation). Tajima's *D* [55] and Fu's *Fs* [56] neutrality test statistics were also estimated. Under assumptions of neutrality, negative values indicate a signature of population expansion. Fu's *Fs* is less conservative than Tajima's *D*, and is expected to be more sensitive to large population expansions (indicated by highly significant negative values). Mismatch distributions [30] and φ_ST_ (using a substitution rate model determined by MODELTEST [50]) were estimated using ARLEQUIN v. 3.01 [57]. The validity of the estimated stepwise expansion model was tested using the sum of squared deviations (SSD) between the observed and the expected mismatch as a test statistic. Both the mismatch distribution and neutrality test statistics operate under infinite site assumptions which may not hold, particularly for the large number of haplotypes identified within the VLC sample. Thus, these estimates were derived for MQ and VLC to see whether results were concordant with demographic model selection implemented in BEAST.

To understand changes in VLC population size through time, FLUCTUATE [31] analyses was implemented using 20 short chains of 1,000 steps each, and five long chains of 20,000 steps were used to determine parameters for the production runs. Production runs were implemented using 20 short chains of 8,000 steps each, and 10 long chains of 50,000 steps. The program was run multiple times to ensure concordance of parameter estimates. Haplotype richness for VLC was measured in relation to MQ (*n* = 49) using Rarefactor (http://www2.biology.ualberta.ca/jbrzusto/rarefact.php). An Isolation-with-Migration model was implemented in IM [29] to estimate effective population sizes, time of splitting, and migration rates between the VLC and MQ populations. All IM analyses used a flat prior (0–10) for m (migration) and t (divergence time), and changes were allowed in population size incorporated into the IM model. To facilitate these analyses, the substitution rate estimated in BEAST was incorporated. This was converted to a rate per generation based on a 4-year generation time for *N*e estimates (Table S2). Three independent replicates of each pairwise comparison were performed, with the Markov chains run for 100,000,000 generations, after discarding a 10% burn-in. To ensure convergence, simulations were run until the smallest ESS estimates were greater than 200, and update rates were greater than 20%. Although the basic IM model cannot account for the sizes of founding populations, the parameter ‘*s*’ controls for this [21], providing for the proportion of the ancestral population that founds the descendant populations. In this way, expansion or contraction can be modelled. A uniform prior distribution for *s* between 0.5 and 1.0 was set (after a similar analysis in [21]) to ensure that the estimates were robust to different patterns of expansion (data not shown). IM results were concordant with either uninformative (0–1) or hard (0.5–1) priors on *s*.

## Supporting Information

Figure S1 BEAST output of southern elephant seal substitution rate estimated from 223 directly radiocarbon dated samples from Victoria Land Coast (VLC). Radiocarbon dates were converted to calendar years.
(1.49 MB TIF)

Figure S2 Isolation-with-migration (IM) output of population genetic parameters for the Macquarie Island (MQ), Victoria Land Coast (VLC) and Ancestral “populations.” See Table S2 for text version of IM parameter estimate distributions.
(8.75 MB TIF)

Figure S3 BEAST output of Time-to-Most-Recent-Common-Ancestor (TMRCA) estimates (YBP). For: all modern populations grouped (besides Macquarie Island) (blue), Macquarie Island (MQ - yellow), and Victoria Land Coast (VLC - red).
(1.49 MB TIF)

Figure S4 BEAST output of southern elephant seal substitution rate estimated from 223 randomized radiocarbon dated samples from Victoria Land Coast (VLC). Radiocarbon dates were converted to calendar years. Dates were randomly assigned to sequences to assess whether the non-randomized ages and sequences were rate informative. These analyses were repeated three times, and all randomized rate estimates were highly skewed to zero.
(1.99 MB TIF)

Table S1 Sample location and age.
(0.32 MB DOC)

Table S2 Isolation-with-migration (IM) output of population genetic parameters for the Macquarie Island (1), Victoria Land Coast (2) and Ancestral (a) “populations.” See Figure S2 for graphic depiction of IM parameter estimate distributions.
(0.04 MB DOC)

Table S3 Parameter comparisons against chronology subsets for VLC. Φ_ST_ = pairwise estimate between MQ and VLC “subset.” % TMRCA = subset age interval (%) relative to the time-to-most-recent-common-ancestor of the VLC sample (estimated at 11,740 YBP in BEAST).
(0.03 MB DOC)
