# Supplementary figures and images for: Rapid Response of a Marine Mammal Species to Holocene Climate and Habitat Change
Source: PLoS Genet. 2009 Jul 10;5(7):e1000554. doi: 10.1371/journal.pgen.1000554 (PMC2700269; doi:10.1371/journal.pgen.1000554)

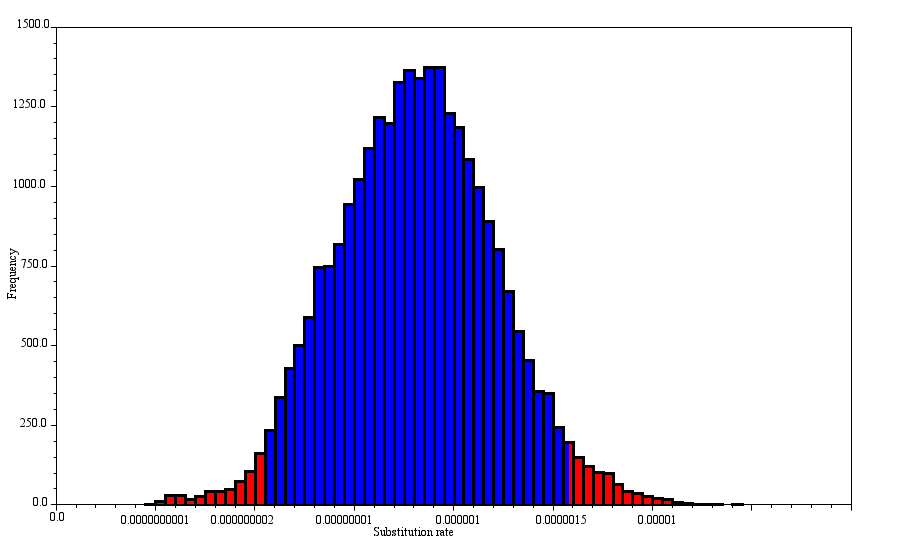

Supplement: Figure S1 — BEAST output of southern elephant seal substitution rate estimated from 223 directly radiocarbon dated samples from Victoria Land Coast (VLC). Radiocarbon dates were converted to calendar years. (1.49 MB TIF) [file pgen.1000554.s001.tif]

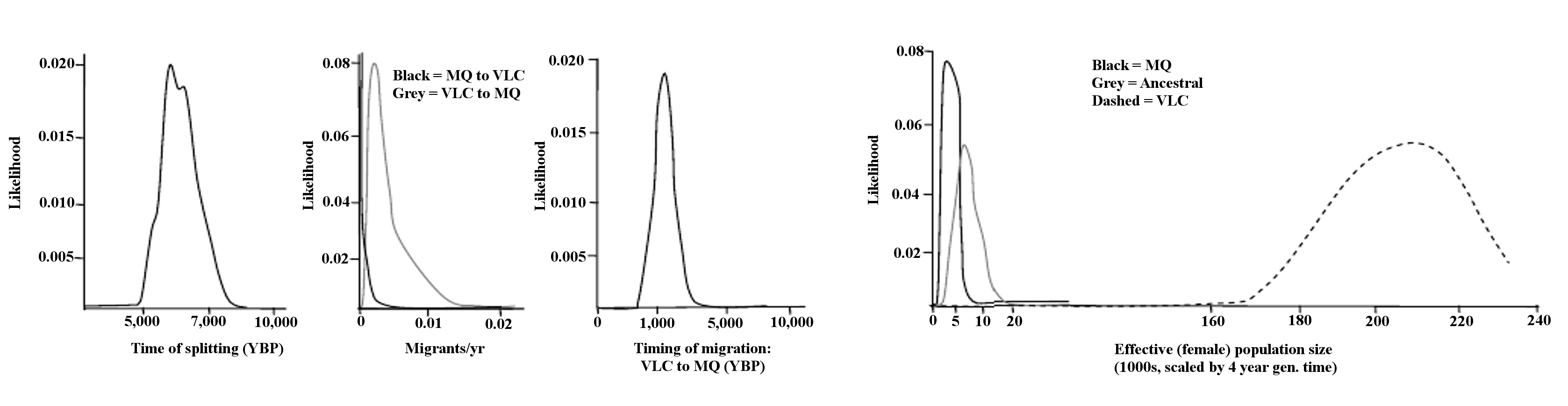

Supplement: Figure S2 — Isolation-with-migration (IM) output of population genetic parameters for the Macquarie Island (MQ), Victoria Land Coast (VLC) and Ancestral “populations.” See Table S2 for text version of IM parameter estimate distributions. (8.75 MB TIF) [file pgen.1000554.s002.tif]

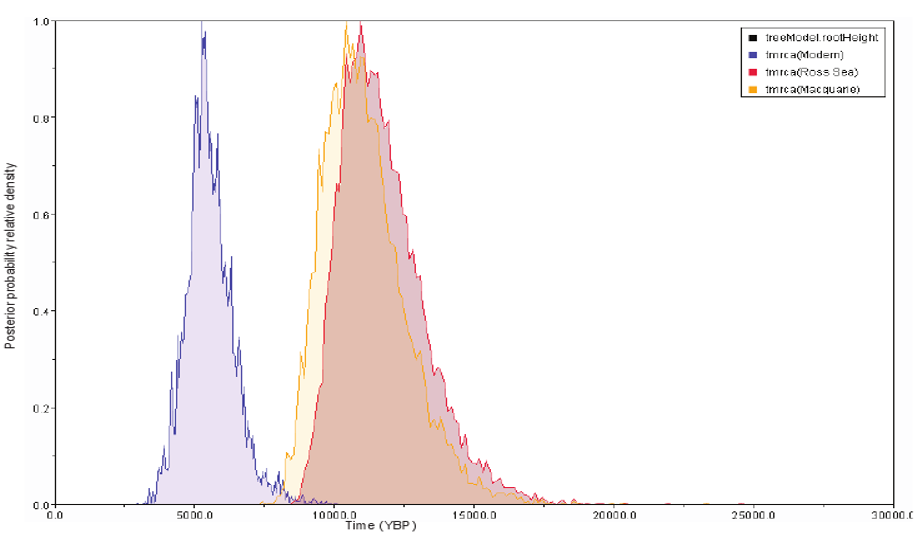

Supplement: Figure S3 — BEAST output of Time-to-Most-Recent-Common-Ancestor (TMRCA) estimates (YBP). For: all modern populations grouped (besides Macquarie Island) (blue), Macquarie Island (MQ - yellow), and Victoria Land Coast (VLC - red). (1.49 MB TIF) [file pgen.1000554.s003.tif]

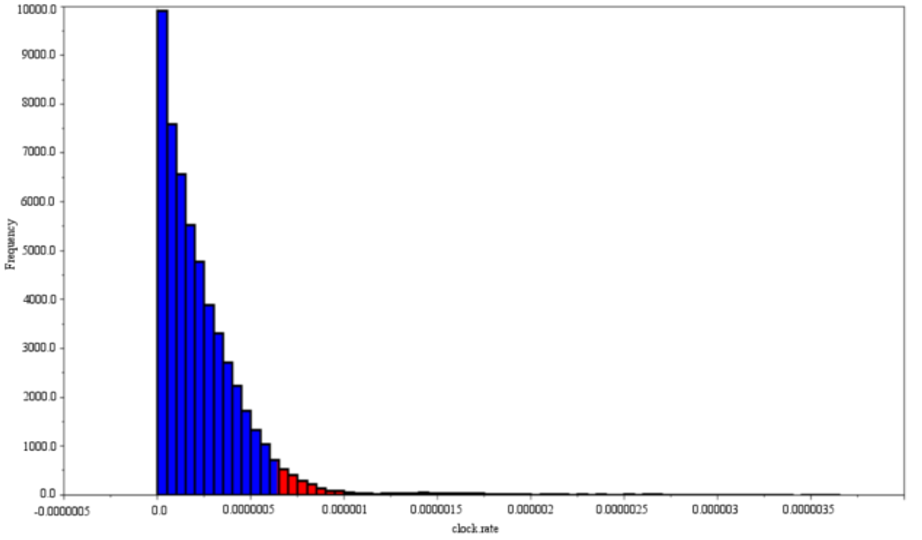

Supplement: Figure S4 — BEAST output of southern elephant seal substitution rate estimated from 223 randomized radiocarbon dated samples from Victoria Land Coast (VLC). Radiocarbon dates were converted to calendar years. Dates were randomly assigned to sequences to assess whether the non-randomized ages and sequences were rate informative. These analyses were repeated three times, and all randomized rate estimates were highly skewed to zero. (1.99 MB TIF) [file pgen.1000554.s004.tif]
