# Supplementary material for: Rapid Response of a Marine Mammal Species to Holocene Climate and Habitat Change
Source: PLoS Genet. 2009 Jul 10;5(7):e1000554. doi: 10.1371/journal.pgen.1000554 (PMC2700269; doi:10.1371/journal.pgen.1000554)
Supplement: Table S1 — Sample location and age. (0.32 MB DOC) [file pgen.1000554.s005.doc]

| **Sample** | **Marine reservoir corrected calendar age** | **Location** | **Latitude**  **(South)** | **Longitude**  **(East)** | **Radiometric age** | **Error** |
| --- | --- | --- | --- | --- | --- | --- |
| 00-11 | 772 | South Bay | 74.91392 | 163.7145 | 2089 | 142 |
| 00-33 | 768 | South Bay | 74.91392 | 163.718 | 2083 | 144.5 |
| 00-50 | 6377 | Seaview Bay | 74.89473 | 163.7 | 6740 | 145.5 |
| 00-54 | 2162 | Marble Point | 77.43 | 163.799 | 3258 | 106 |
| 01-226 | 7087 | South Bay | 74.91 | 163.6766 | 7365 | 153 |
| 01-227 | 1125 | South Bay | 74.91307 | 163.7165 | 2469 | 153.5 |
| 01-235d | 4663 | South Bay | 74.91392 | 163.7143 | 5276 | 166 |
| 01-244b | 4047 | Seaview Bay | 74.89342 | 163.7411 | 4817 | 178 |
| 01-255b | 1579 | Depot Is. Peninsula | 76.69947 | 162.94 | 2735 | 97.5 |
| 01-257 | 2586 | Depot Is. Peninsula | 76.69947 | 162.9415 | 3572 | 137 |
| 01-258 | 2197 | Depot Is. Peninsula | 76.69947 | 162.9415 | 3290 | 97 |
| 01-B1 | 321 | South Bay | 74.91875 | 163.6765 | 1566 | 60 |
| 01-S5 | 572 | Seaview Bay | 74.9 | 163.730 | 1884 | 47.5 |
| 05-01A | 558 | Dunlop I. | 77.23177 | 163.4683 | 1861 | 47.5 |
| 05-100B | 908 | Spike Cape | 77.30515 | 163.5711 | 2256 | 170 |
| 05-102 | 1320 | Spike Cape | 77.30727 | 163.5639 | 2501 | 80 |
| 05-106B | 2226 | Spike Cape | 77.30383 | 163.526 | 3330 | 94 |
| 05-112A | 2431 | Kolich Pt | 77.36203 | 163.5369 | 3491 | 109 |
| 05-116B | 2797 | Spike Cape | 77.30768 | 163.5311 | 3727 | 133.5 |
| 05-119B | 898 | Spike Cape | 77.30782 | 163.539 | 2248 | 160.5 |
| 05-121A | 2421 | Kolich Pt | 77.36203 | 163.5369 | 3481 | 108 |
| 05-127A | 2216 | Gneiss Pt | 77.39618 | 163.651 | 3318 | 95.5 |
| 05-127G | 2414 | Gneiss Pt. | 77.39618 | 163.651 | 3420 | 168 |
| 05-130 | 2339 | Gneiss Pt | 77.39618 | 163.651 | 3411 | 99 |
| 05-131 | 6242 | Gneiss Pt | 77.39465 | 163.7144 | 6613 | 146 |
| 05-132A | 4939 | Gneiss Pt | 77.39415 | 163.7097 | 5520 | 126.5 |
| 05-132D | 3418 | Gneiss Point | 77 23.649 | 163 42.584 | 4320 | 152.5 |
| 05-138A | 4074 | Marble Pt | 77 26.100 | 163 46.677 | 4610 | 99 |
| 05-145A | 2046 | Bernacchi Bay | 77.46233 | 163.7522 | 3190 | 99.5 |
| 05-145J | 2399 | Bernacchi Bay | 77 27.740 | 163 45.131 | 3460 | 105.5 |
| 05-146 | 1147 | Bernacchi Bay | 77.46397 | 163.7594 | 2499 | 158 |
| 05-15 | 2260 | Dunlop I. | 77.2355 | 163.508 | 3385 | 102.5 |
| 05-155A | 938 | Marble Point | 77.4297 | 163.7547 | 2214 | 164 |
| 05-157 | 851 | Marble Pt | 77 25.859 | 163 45.725 | 2200 | 154 |
| 05-158E | 823 | Marble Point | 77.43098 | 163.7621 | 2173 | 149.5 |
| 05-159C | 1438 | Marble Point | 77.43098 | 163.7621 | 2631 | 94 |
| 05-22 | 2355 | Dunlop I. | 77.23147 | 163.4721 | 3424 | 68.5 |
| 05-39B | 1958 | Dunlop I. | 77.23538 | 163.4851 | 3110 | 91.5 |
| 05-42B | 3076 | Dunlop I. | 77.23407 | 163.4781 | 3980 | 145.5 |
| 05-44B | 3383 | Dunlop I. | 77.23527 | 163.5034 | 4283 | 155.5 |
| 05-49 | 2300 | Dunlop I. | 77.23617 | 163.5162 | 3400 | 106.5 |
| 05-26 | 2437 | Dunlop I. | 77.23147 | 163.4721 | 3495 | 113 |
| 05-54D | 1128 | Dunlop I. | 77.23733 | 163.5234 | 2474 | 155 |
| 05-59B | 3615 | Dunlop I. | 77.2326 | 163.4843 | 4488 | 160 |
| 05-70 | 813 | C. Roberts | 77.03365 | 163.1605 | 2159 | 147 |
| 05-71A | 1621 | Cape Roberts | 77.03407 | 163.1603 | 2790 | 91.5 |
| 05-73 | 934 | C. Roberts | 77.03355 | 163.1564 | 2274 | 163.5 |
| 05-74A | 1379 | C. Roberts | 77.03437 | 163.1555 | 2568 | 81 |
| 05-75 | 4955 | Cape Roberts | 77.03533 | 163.1541 | 5470 | 178 |
| 05-76A | 1602 | C. Roberts | 77.0341 | 163.0341 | 2764 | 94.5 |
| 05-80B | 888 | C. Roberts | 77.0341 | 163.1731 | 2233 | 159 |
| 05-82A | 3035 | Cape Roberts | 77.03763 | 163.166 | 4010 | 163 |
| 05-84A | 879 | Cape Roberts | 77.03497 | 163.1827 | 2220 | 156.5 |
| 05-87A | 1420 | Depot Island P. | 76.69947 | 162.9416 | 2606 | 83.5 |
| 05-88c | 3020 | Depot Island P. | 76.69933 | 162.9435 | 3940 | 150.5 |
| 05-89B | 2572 | Depot Island P. | 76.69985 | 162.9382 | 3556 | 136 |
| 05-93A | 1076 | Cape Roberts | 77.03613 | 163.168 | 2400 | 155 |
| 05-96E | 4953 | Cape Roberts | 77.03533 | 163.1541 | 5467 | 179.5 |
| 05-98A | 776 | Spike Cape | 77.30558 | 163.57 | 2098 | 141.5 |
| S06-13A | 513 | Seaview Bay | 74.89548 | 163.7347 | 1810 | 44 |
| 94-146 | 1663 | Cape Roberts | 77.03533 | 163.16 | 2820 | 109 |
| 95-331 | 842 | Cape Roberts | 77.03365 | 163.1606 | 2190 | 161 |
| 95-342 | 3076 | Cape Roberts | 77.03613 | 163.168 | 3977 | 163 |
| 95-346 | 958 | Cape Roberts | 77.03365 | 163.1605 | 2295 | 169.5 |
| 95-350 | 1713 | Cape Roberts | 77.0335 | 163.15 | 2864 | 103 |
| 95-353 | 1503 | Dunlop Is. | 77.23527 | 163.499 | 2692 | 102.5 |
| 95-361 | 2321 | Dunlop Is. | 77.2326 | 163.5 | 3410 | 118.5 |
| 95-364 | 2258 | Dunlop Is. | 77.23 | 163.5 | 3380 | 111.5 |
| 95-388 | 1565 | Cape Roberts | 77.0335 | 163.15 | 2725 | 100 |
| 95-393 | 2575 | Dunlop Is. | 77.2365 | 163.517 | 3560 | 139 |
| 95-394C | 1406 | Dunlop Is. | 77.2365 | 163.517 | 2593 | 116.5 |
| 95-395 | 2358 | Dunlop Is. | 77.2365 | 163.517 | 3425 | 117.5 |
| 95-398 | 2225 | Dunlop Is. | 77.23611 | 163.4861 | 3330 | 96.5 |
| 95-399 | 2078 | Dunlop Is. | 77.23611 | 163.4861 | 3210 | 122.5 |
| 95-400 | 1744 | Dunlop Is. | 77.23611 | 163.4861 | 2890 | 113 |
| 95-437 | 1099 | Marble Point | 77.43 | 163.85 | 2430 | 157 |
| CB06-01B | 1584 | Cape Bird | 77.24238 | 166.3821 | 2740 | 97 |
| CB06-02 | 2073 | Cape Bird | 77.24382 | 166.3813 | 3040 | 89.5 |
| CB06-03 | 1607 | Cape Bird | 77.24275 | 166.3826 | 2770 | 96 |
| CB06-04 | 1584 | Cape Bird | 77.24237 | 166.3822 | 2740 | 94.5 |
| CB06-09 | 426 | Cape Bird | 77.24393 | 166.3666 | 1690 | 60 |
| CB06-13 | 1613 | Cape Bird | 77.24302 | 166.3773 | 2780 | 93.5 |
| CB06-14 | 1423 | Cape Bird | 77.24305 | 166.3773 | 2610 | 81.5 |
| CB06-16 | 1583 | Cape Bird | 77.243 | 166.3775 | 2740 | 100 |
| CB06-17 | 1573 | Cape Bird | 77.24283 | 166.3792 | 2730 | 96.5 |
| CB06-18 | 1360 | Cape Bird | 77.2428 | 166.3801 | 2550 | 79 |
| CB06-19 | 1148 | Cape Bird | 77.24262 | 166.3803 | 2500 | 156.5 |
| CB06-20 | 1457 | Cape Bird | 77.24263 | 166.3804 | 2650 | 86.5 |
| CB06-22 | 1850 | Cape Bird | 77.24278 | 166.3821 | 2980 | 93.5 |
| CB06-25 | 1468 | Cape Bird | 77.24433 | 166.367 | 2660 | 95 |
| CR06-01 | 3114 | Cape Ross | 76.72952 | 163.0013 | 4070 | 167.5 |
| CR06-02C | 3363 | Cape Ross | 76.7295 | 163.0013 | 4270 | 156.5 |
| CR06-03B | 3538 | Cape Ross | 76.72953 | 163.0013 | 4430 | 151 |
| CR06-07B | 3315 | Cape Ross | 76.7296 | 163.0013 | 4240 | 162 |
| CR06-09 | 3294 | Cape Ross | 76.72955 | 163.0015 | 4220 | 158 |
| CR06-10B | 3465 | Cape Ross | 76.72987 | 163.0014 | 4350 | 152.5 |
| CRS7 | 4770 | Cape Roberts | 77.03822 | 163.1627 | 5460 | 175 |
| DI06-01 | 5186 | Dunlop Island | 77.23515 | 163.4801 | 5730 | 144 |
| DI06-02 | 5367 | Dunlop Island | 77.23515 | 163.4801 | 5830 | 113.5 |
| DI06-03 | 5172 | Dunlop Island | 77.23512 | 163.4811 | 5710 | 140.5 |
| DI06-05A | 4523 | Dunlop Island | 77.23532 | 163.48 | 4940 | 101 |
| DI06-07 | 5307 | Dunlop Island | 77.23528 | 163.4806 | 5770 | 129 |
| DI06-09 | 5068 | Dunlop Island | 77.23515 | 163.4789 | 5600 | 139.5 |
| DI06-10 | 3198 | Dunlop Island | 77.23212 | 163.4731 | 4140 | 157.5 |
| DIS-1 | 2963 | Dunlop I | 77.23342 | 163.4712 | 3892 | 142.5 |
| II06-03 | 814 | South Bay | 74.91255 | 163.7108 | 2160 | 149.5 |
| II06-04 | 5174 | South Bay | 74.91767 | 163.6862 | 5710 | 131.5 |
| II06-05B | 2540 | South Bay | 74.91903 | 163.7079 | 3540 | 161.5 |
| II06-08 | 5173 | South Bay | 74.917 | 163.7158 | 5710 | 134.5 |
| II06-100 | 1490 | Seaview Bay | 74.89468 | 163.725 | 2680 | 91.5 |
| II06-101C | 4275 | South Bay | 74.9134 | 163.7155 | 4740 | 96 |
| II06-103 | 4589 | South Bay | 74.91393 | 163.7126 | 5210 | 152.5 |
| II06-104B | 4254 | South Bay | 74.91392 | 163.7143 | 4730 | 97 |
| II06-106 | 3315 | Unnamed Cove | 74.93745 | 163.6791 | 4240 | 158 |
| II06-107 | 270 | Unnamed Cove | 74.93745 | 163.6791 | 4240 | 158 |
| II06-107B | 1144 | Unnamed Cove | 74.93615 | 163.7065 | 1530 | 90.5 |
| 05-108C | 2804 | Spike Cape | 77.30683 | 163.5297 | 3733 | 157 |
| II06-109E | 3530 | Whisker Cove | 74.92953 | 163.7097 | 4420 | 150 |
| II06-110A | 3198 | Whisker Cove | 74.92945 | 163.7097 | 4140 | 157.5 |
| II06-112A | 4504 | Whisker Cove | 74.92802 | 163.7096 | 4920 | 95.5 |
| II06-113 | 3035 | Whisker Cove | 74.92808 | 163.709 | 4010 | 163 |
| II06-114 | 2845 | Whisker Cove | 74.92797 | 163.7094 | 3790 | 123 |
| II06-115 | 952 | Whisker Cove | 74.9283 | 163.7084 | 2290 | 164 |
| II06-116C | 4589 | South Bay | 74.91995 | 163.7094 | 5210 | 152.5 |
| II06-121D | 1076 | Seaview Bay | 74.8952 | 163.7273 | 2400 | 155 |
| II06-122D | 1644 | Seaview Bay | 74.8957 | 163.727 | 2810 | 96 |
| II06-124 | 2181 | Seaview Bay | 74.89473 | 163.7275 | 3270 | 97.5 |
| II06-125C | 1798 | Seaview Bay | 74.89575 | 163.7272 | 2940 | 92.5 |
| II06-128 | 1341 | Seaview Bay | 74.89595 | 163.7273 | 2530 | 76 |
| II06-13 | 777 | South Bay | 74.91362 | 163.7155 | 2100 | 140.5 |
| II06-134 | 1076 | Seaview Bay | 74.89547 | 163.728 | 2400 | 155 |
| II06-135 | 1106 | Seaview Bay | 74.89542 | 163.7264 | 2440 | 153.5 |
| II06-139E | 1837 | Seaview Bay | 74.89565 | 163.7273 | 3010 | 91 |
| II06-149C | 1584 | South Bay | 74.91833 | 163.7105 | 2740 | 97 |
| II06-152 | 1755 | South Bay | 74.91855 | 163.7089 | 2890 | 95.5 |
| II06-155A | 2829 | South Bay | 74.91982 | 163.7093 | 3770 | 122.5 |
| II06-158 | 437 | South Bay | 74.92002 | 163.7116 | 1710 | 66 |
| II06-161B | 4345 | South Bay | 74.91437 | 163.7142 | 4810 | 97 |
| II06-164B | 868 | South Bay | 74.91105 | 163.7187 | 2210 | 155 |
| II06-168 | 1869 | South Bay | 74.9117 | 163.7114 | 3030 | 91 |
| II06-169A | 2009 | South Bay | 74.91213 | 163.7094 | 3150 | 92.5 |
| II06-17 | 1651 | South Bay | 74.91352 | 163.716 | 2810 | 111 |
| II06-170 | 2151 | South Bay | 74.912 | 163.7085 | 3250 | 114.5 |
| II06-171 | 5173 | South Bay | 74.91247 | 163.7066 | 5710 | 134.5 |
| II06-172 | 4817 | South Bay | 74.91205 | 163.7069 | 5400 | 185 |
| II06-173A | 782 | South Bay | 74.91207 | 163.7095 | 2110 | 140.5 |
| II06-175B | 5179 | South Bay | 74.91277 | 163.7069 | 5720 | 134.5 |
| II06-179 | 900 | South Bay | 74.91215 | 163.7102 | 2250 | 161.5 |
| II06-183 | 880 | South Bay | 74.91228 | 163.7105 | 2220 | 165 |
| II06-185 | 3294 | South Bay | 74.91207 | 163.7099 | 4220 | 158 |
| II06-18A | 3128 | South Bay | 74.91392 | 163.7155 | 4080 | 161 |
| II06-19 | 684 | South Bay | 74.91392 | 163.7159 | 2010 | 139.5 |
| II06-190 | 813 | South Bay | 74.9122 | 163.7114 | 2160 | 145.5 |
| II06-193 | 941 | South Bay | 74.9119 | 163.7111 | 2280 | 163.5 |
| II06-23 | 3008 | South Bay | 74.9141 | 163.7153 | 3930 | 144.5 |
| II06-26A | 2889 | South Bay | 74.91443 | 163.7154 | 3840 | 131.5 |
| II06-28 | 3144 | South Bay | 74.9142 | 163.7153 | 4090 | 162 |
| II06-30 | 1731 | South Bay | 74.91407 | 163.7167 | 2880 | 94.5 |
| II06-34 | 1702 | South Bay | 74.91477 | 163.7167 | 2850 | 101.5 |
| II06-49 | 2369 | South Bay | 74.91442 | 163.7142 | 3430 | 108.5 |
| II06-53 | 2681 | South Bay | 74.91478 | 163.7134 | 3660 | 144.5 |
| II06-60 | 1393 | South Bay | 74.91365 | 163.7128 | 2580 | 82 |
| II06-82E | 1882 | Seaview Bay | 74.89395 | 163.7429 | 3040 | 96 |
| II06-85 | 749 | Seaview Bay | 74.8936 | 163.7421 | 2050 | 142 |
| II06-86C | 2009 | Seaview Bay | 74.89347 | 163.741 | 3150 | 95.5 |
| II06-87B | 3284 | Seaview Bay | 74.89298 | 163.7402 | 4210 | 158.5 |
| II06-89 | 3284 | Seaview Bay | 74.8931 | 163.741 | 4210 | 158.5 |
| II06-96A | 1958 | Seaview Bay | 74.89342 | 163.7411 | 3110 | 91.5 |
| MZ06-05 | 749 | Edmonson Pt | 74.3262 | 165.1183 | 2050 | 142 |
| MZ06-12 | 581 | Edmonson Pt | 74.32608 | 165.118 | 1900 | 47 |
| MZ06-13 | 570 | Edmonson Pt | 74.32627 | 165.1161 | 1880 | 47.5 |
| MZ06-19 | 581 | Edmonson Pt | 74.32655 | 165.1183 | 1900 | 47 |
| MZ06-22 | 359 | Edmonson Pt | 74.32597 | 165.1177 | 1620 | 59 |
| MZ06-26 | 564 | Edmonson Pt | 74.32628 | 165.1162 | 1870 | 47.5 |
| MZ06-30 | 851 | Edmonson Pt | 74.3259 | 165.1198 | 2200 | 157 |
| MZ06-33 | 652 | Edmonson Pt | 74.32575 | 165.1204 | 1990 | 53.5 |
| MZ06-34 | 570 | Edmonson Pt | 74.32522 | 165.1181 | 1880 | 47.5 |
| MZ06-36 | 415 | Edmonson Pt | 74.32643 | 165.1165 | 1680 | 61.5 |
| MZ06-40A | 1837 | Gondwana | 74.63458 | 164.2253 | 3010 | 91 |
| MZ06-41 | 870 | Gondwana | 74.6344 | 164.2253 | 2210 | 158.5 |
| MZ06-44 | 586 | Gondwana | 74.63448 | 164.2264 | 1910 | 45.5 |
| MZ06-46 | 1868 | Gondwana | 74.63453 | 164.2252 | 3030 | 99.5 |
| MZ06-49 | 2997 | Gondwana | 74.63425 | 164.2248 | 3920 | 144 |
| MZ06-52 | 4236 | Gondwana | 74.63423 | 164.2245 | 4710 | 102.5 |
| MZ06-54 | 3089 | Gondwana | 74.63413 | 164.2237 | 3990 | 145.5 |
| MZ06-56 | 4191 | Gondwana | 74.63415 | 164.2234 | 4690 | 104 |
| MZ06-58 | 4973 | Gondwana | 74.63375 | 164.222 | 5500 | 161.5 |
| MZ06-59 | 5162 | Gondwana | 74.6337 | 164.2221 | 5690 | 134.5 |
| MZ06-60 | 4744 | Gondwana | 74.6334 | 164.2216 | 5350 | 183 |
| MZ06-61 | 5022 | Gondwana | 74.63375 | 164.2226 | 5590 | 117.5 |
| MZ06-63 | 4853 | Gondwana | 74.63358 | 164.2227 | 5420 | 179.5 |
| MZ06-65 | 581 | Adelie Cove | 74.77947 | 163.9792 | 1900 | 48.5 |
| MZ06-69 | 570 | Adelie Cove | 74.77918 | 163.9785 | 1880 | 46 |
| MZ06-71 | 602 | Adelie Cove | 74.77965 | 163.9798 | 1940 | 47.5 |
| MZ06-72 | 591 | Adelie Cove | 74.7796 | 163.9795 | 1920 | 52.5 |
| S06-01 | 968 | South Bay | 74.91272 | 163.7145 | 2300 | 146 |
| S06-02 | 766 | South Bay | 74.91313 | 163.7168 | 2080 | 139.5 |
| S06-03 | 520 | South Bay | 74.91337 | 163.7164 | 1820 | 44.5 |
| S06-04 | 1621 | South Bay | 74.91263 | 163.7178 | 2790 | 91.5 |
| S06-06 | 3035 | South Bay | 74.91578 | 163.7146 | 4010 | 162 |
| S06-07 | 478 | South Bay | 74.92008 | 163.7143 | 1760 | 44.5 |
| S06-08 | 3187 | South Bay | 74.9139 | 163.713 | 4130 | 160 |
| S06-09 | 1069 | South Bay | 74.91255 | 163.7108 | 2390 | 155.5 |
| S06-10 | 436 | South Bay | 74.91872 | 163.6768 | 1700 | 58 |
| S06-11 | 755 | Seaview Bay | 74.89518 | 163.732 | 2060 | 139.5 |
| S06-12 | 493 | Seaview Bay | 74.89535 | 163.7337 | 1780 | 47 |
| S06-13B | 513 | Seaview Bay | 74.89548 | 163.7347 | 1810 | 44 |
| S06-14 | 506 | Seaview Bay | 74.89515 | 163.7353 | 1800 | 45 |
| S06-15 | 720 | Seaview Bay | 74.89502 | 163.7345 | 2030 | 130 |
| S06-17 | 1631 | Seaview Bay | 74.89508 | 163.7346 | 2800 | 92.5 |
| S06-18 | 2038 | South Bay | 74.91335 | 163.7108 | 3180 | 93 |
| S06-19 | 2997 | South Bay | 74.91452 | 163.713 | 3920 | 144 |
| S06-20 | 1439 | South Bay | 74.91537 | 163.7163 | 2630 | 81 |
| S06-21 | 1081 | South Bay | 74.91292 | 163.7174 | 2410 | 153.5 |
| S06-22 | 1125 | Seaview Bay | 74.89577 | 163.7301 | 2470 | 154.5 |
| S06-23 | 1854 | Seaview Bay | 74.8954 | 163.7319 | 3020 | 89 |
| S06-30 | 631 | Seaview Bay | 74.89357 | 163.7286 | 1970 | 41.5 |
| S06-31 | 1677 | Seaview Bay | 74.8955 | 163.7246 | 2830 | 100 |
| S06-32 | 3273 | South Bay | 74.91388 | 163.7153 | 4200 | 157.5 |
| S06-33 | 3104 | South Bay | 74.91363 | 163.7131 | 4100 | 109.5 |
| S06-36 | 1099 | South Bay | 74.91335 | 163.7164 | 2430 | 153.5 |
| S06-49 | 3226 | South Bay | 74.91393 | 163.7139 | 4170 | 157.5 |
| Seal-6 | 562 | South Bay | 74.92007 | 163.7143 | 1866 | 49 |
