# Supplementary material for: Rapid Response of a Marine Mammal Species to Holocene Climate and Habitat Change
Source: PLoS Genet. 2009 Jul 10;5(7):e1000554. doi: 10.1371/journal.pgen.1000554 (PMC2700269; doi:10.1371/journal.pgen.1000554)
Supplement: Table S2 — Isolation-with-migration (IM) output of population genetic parameters for the Macquarie Island (1), Victoria Land Coast (2) and Ancestral (a) “populations.” See Figure S2 for graphic depiction of IM parameter estimate distributions. (0.04 MB DOC) [file pgen.1000554.s006.doc]

| **Value** | **q1** | **q2** | **qa** | **m1** | **m2** | **t** |
| --- | --- | --- | --- | --- | --- | --- |
| **Minbin** | 5.310 | 678.927 | 15.930 | 0.005 | 0.005 | 1.155 |
| **Maxbin** | 125.165 | 1516.397 | 324.255 | 2.150 | 1.555 | 2.985 |
| **HiPt** | 34.281 | 1117.323 | 67.588 | 0.450 | 0.005 | 1.965 |
| **HiSmth** | 34.165 | 1118.755 | 67.588 | 0.445 | 0.005 | 1.960 |
| **Mean** | 34.895 | 1116.572 | 68.467 | 0.440 | 0.005 | 1.950 |
| **95Lo** | 12.785 | 926.389 | 45.385 | 0.060 | 0.005 | 1.560 |
| **95Hi** | 63.555 | 1401.611 | 126.745 | 1.850 | 0.205 | 2.570 |
| **HPD90Lo** | 13.076 | 957.477 | 47.495 | 0.070 | 0.005 | 1.629 |
| **HPD90Hi** | 60.994 | 1363.598 | 116.326 | 1.790 | 0.115 | 2.444 |
| **Tail?** | complete | complete | complete | complete | complete | complete |
| **ESS** | 348 | 294 | 203 | 205 | 230 | 230 |

**Table S2. Isolation-with-migration (IM) output of population genetic parameters for the Macquarie Island (1), Victoria Land Coast (2) and Ancestral (a) ‘populations’.** See Figure S2 for graphic depiction of IM parameter estimate distributions.
