# Supplementary material for: Rapid Response of a Marine Mammal Species to Holocene Climate and Habitat Change
Source: PLoS Genet. 2009 Jul 10;5(7):e1000554. doi: 10.1371/journal.pgen.1000554 (PMC2700269; doi:10.1371/journal.pgen.1000554)
Supplement: Table S3 — Parameter comparisons against chronology subsets for VLC. ΦST = pairwise estimate between MQ and VLC “subset.” % TMRCA = subset age interval (%) relative to the time-to-most-recent-common-ancestor of the VLC sample (estimated at 11,740 YBP in BEAST). (0.03 MB DOC) [file pgen.1000554.s007.doc]

|  | **Age** | **%TMRCA** | ***N*** | ***S*** | ***h*** | ***Fs*** | ***D*** | **φ_ST_** |
| --- | --- | --- | --- | --- | --- | --- | --- | --- |
| **MQ** | 0 | N/A | 48 | 23 | 16 | -0.11; *P*=0.13 | 0.88; *P*>0.10 | N/A |
| **VLC All** | 270-7,087 | 58.1 | 223 | 91 | 177 | -323.00; *P*<0.01 | -1.66; *P*>0.05 | 0.17 |
| **VLC 1000** | 938-1,148 | 1.8 | 16 | 37 | 16 | -8.707; *P*<0.01 | -0.63; *P*>0.10 | 0.13 |
| **VLC 2000** | 1,958-2,300 | 2.9 | 18 | 28 | 16 | -7.767; *P*<0.01 | -0.72; *P*>0.10 | 0.16 |
| **VLC 3000** | 2,963-3,363 | 3.4 | 23 | 36 | 23 | -17.72; *P*<0.01 | -0.80; *P*>0.10 | 0.19 |
| **VLC 4000** | 4,047-4,589 | 4.6 | 11 | 20 | 11 | -5.625; *P*<0.01 | -0.23; *P*>0.10 | 0.23 |
| **VLC 5000** | 4,939-5,367 | 3.6 | 16 | 24 | 14 | -5.921; *P*<0.01 | -0.42; *P*>0.10 | 0.22 |

**Table S3: Parameter comparisons against chronology subsets for VLC.** φ_ST_ = pairwise estimate between MQ and VLC ‘subset’. % TMRCA = subset age interval (%) relative to the time-to-most-recent-common-ancestor of the VLC sample (estimated at 11,740 YBP in BEAST)
